# Randomized clinical trial to evaluate the effect of fecal microbiota transplant for initial *Clostridium difficile* infection in intestinal microbiome

**DOI:** 10.1371/journal.pone.0189768

**Published:** 2017-12-20

**Authors:** Adrián Camacho-Ortiz, Eva María Gutiérrez-Delgado, Jose F. Garcia-Mazcorro, Soraya Mendoza-Olazarán, Adrián Martínez-Meléndez, Laura Palau-Davila, Simon D. Baines, Héctor Maldonado-Garza, Elvira Garza-González

**Affiliations:** 1 Coordinación de Epidemiología Hospitalaria, Hospital Universitario “Dr. José Eleuterio González”, Universidad Autónoma de Nuevo León, Monterrey, Nuevo Leon, Mexico; 2 Servicio de Infectología, Hospital Universitario “Dr. José Eleuterio González”, Universidad Autónoma de Nuevo León, Monterrey, Nuevo Leon, Mexico; 3 Laboratorio de Fisiología Digestiva y Motilidad Gastrointestinal, Instituto de Investigaciones Medico-Biológicas, Universidad Veracruzana, Veracruz, México; 4 Servicio de Gastroenterología, Hospital Universitario “Dr. José Eleuterio González”, Universidad Autónoma de Nuevo León, Monterrey, Nuevo Leon, Mexico; 5 Departamento de Microbiología e Inmunología, Facultad de Ciencias Biológicas, Universidad Autónoma de Nuevo León, San Nicolás de los Garza, Nuevo León, Mexico; 6 Department of Biological and Environmental Sciences, School of Life and Medical Sciences, University of Hertfordshire, Hatfield, Hertfordshire, United Kingdom; University Hospital Llandough, UNITED KINGDOM

## Abstract

**Objective:**

The aim of this study was to evaluate the impact of fecal donor-unrelated donor mix (FMT-FURM) transplantation as first-line therapy for *C*. *difficile* infection (CDI) in intestinal microbiome.

**Methods:**

We designed an open, two-arm pilot study with oral vancomycin (250mg every 6 h for 10–14 days) or FMT-FURM as treatments for the first CDI episode in hospitalized adult patients in Hospital Universitario “Dr. Jose Eleuterio Gonzalez”. Patients were randomized by a closed envelope method in a 1: 1 ratio to either oral vancomycin or FMT-FURM. CDI resolution was considered when there was a reduction on the Bristol scale of at least 2 points, a reduction of at least 50% in the number of bowel movements, absence of fever, and resolution of abdominal pain (at least two criteria).

From each patient, a fecal sample was obtained at days 0, 3, and 7 after treatment. Specimens were cultured to isolate *C*. *difficile*, and isolates were characterized by PCR. Susceptibility testing of isolates was performed using the agar dilution method. Fecal samples and FMT-FURM were analyzed by 16S rRNA sequencing.

**Results:**

We included 19 patients; 10 in the vancomycin arm and 9 in the FMT-FURM arm. However, one of the patients in the vancomycin arm and two patients in the FMT-FURM arm were eliminated. Symptoms resolved in 8/9 patients (88.9%) in the vancomycin group, while symptoms resolved in 4/7 patients (57.1%) after the first FMT-FURM dose (*P* = 0.26) and in 5/7 patients (71.4%) after the second dose (*P* = 0.55). During the study, no adverse effects attributable to FMT-FURM were observed in patients.

Twelve isolates were recovered, most isolates carried *tcdB*, *tcdA*, *cdtA*, and *cdtB*, with an 18-bp deletion in *tcdC*. All isolates were resistant to ciprofloxacin and moxifloxacin but susceptible to metronidazole, linezolid, fidaxomicin, and tetracycline. In the FMT-FURM group, the bacterial composition was dominated by Firmicutes, Bacteroidetes, and Proteobacteria at all-time points and the microbiota were remarkably stable over time. The vancomycin group showed a very different pattern of the microbial composition when comparing to the FMT-FURM group over time.

**Conclusion:**

The results of this preliminary study showed that FMT-FURM for initial CDI is associated with specific bacterial communities that do not resemble the donors’ sample.

## Introduction

*Clostridium difficile* is the causative agent of *C*. *difficile* infection (CDI) and accounts for 15–25% of all nosocomial diarrhea cases, with an incidence of 10.4 cases per 1,000 hospital admissions [1, 2]. It is estimated that CDI is responsible for almost half a million infections per year in the United States with an estimated number of deaths of about 30,000 [3]. Hospital-acquired CDI increases the absolute risk of in-hospital mortality over 10% [4].

According to the most recent guidelines [5], the standard treatment for the first episode of CDI or first recurrence of mild or moderate CDI is metronidazole. Severe CDI should be treated with intravenous (IV) metronidazole plus enteral vancomycin [5]. Even though treatment for CDI with oral vancomycin and/or metronidazole is recommended, this approach is associated with a significant therapeutic failure, with reported recurrences up to 30–40% of patients with the first episode and 60% of patients with repeated recurrences[6]. Furthermore, the emergence of the *C*. *difficile* strain NAP1/BI/027 has been associated with more failed treatments[7].

Fecal microbiota transplantation (FMT) represents a therapeutic modality with faster reconstitution of the microbial communities of the colon. It has been used for the treatment of patients with recurrent CDI with an efficacy of 87–91%, even in immunosuppressed patients [8]. FMT has shown to be a safer and more effective treatment than a prolonged vancomycin regimen in recurrent CDI cases, with clinical efficacies of 90% *vs*. 26% in one study [9] and 94% *vs*. 23% in another study [10]. To date, there are no reports on the use of FMT as initial treatment for CDI in hospitalized patients.

The aim of this study was to evaluate the impact of FMT-FURM as first-line therapy of *C*. *difficile* infection in intestinal microbiome.

## Material and methods

### Informed consent

The study was reviewed and approved by the Local Ethics Committee of the School of Medicine of the Universidad Autónoma de Nuevo León (Approval IF14-005). All study participants, or their legal guardian, provided informed written consent before study enrollment. We began recruiting patients once our local ethics committee authorized the research protocol (approved on November 6^th^, 2014). The registry in Clinical trials was performed after the beginning of the protocol.

All ongoing and related trials for this intervention are registered with the ClinicalTrials.gov Identifier: NCT03107169; https://clinicaltrials.gov/ct2/show/NCT03107169?term=camacho+ortiz&rank=1.

### Design of the study, setting and inclusion criteria

We designed an open, per-protocol, two-arm pilot trial using oral vancomycin (250mg every 6 h for 10–14 days) or FMT-FURM as an alternative treatment for the first CDI episode among hospitalized patients. Patients were included in the study from February 2015 to October 2015 in Hospital Universitario “Dr. Jose Eleuterio Gonzalez” in Monterrey, Mexico; a 450-bed teaching hospital with an average of 22,000 annual discharges. Patients’ cases were reviewed and evaluated by the study investigators and research coordinators. Adult patients, *i*.*e*., over 18 years old, who had been hospitalized for any cause and diagnosed with a first CDI episode >48 h after admission were included. None of the patients had a history of CDI or had received prior treatment for the current CDI episode. CDI cases were defined as follows: > 3 bowel movements during the prior 24 h, a Bristol scale > 5, positive test results for *C*. *difficile* toxins A/B detected by either immunoassay (Meridian immunocard *C*. *difficile* toxins A/B) or real-time PCR (Cepheid Xpert *C*. *difficile*/Epi, Cepheid, Sunnyvale CA) in accordance with the manufacturers´ instructions. Patients with a toxic megacolon, suspected or documented intestinal perforation, pregnancy, or the concomitant presence of colon neoplasms were excluded.

### Randomization and study groups

In this pilot study, we proposed a sample size of 9 patients in each group [Power (1-beta) 80%; Significance level (alpha) 5%; Non-inferiority limit, d value 48%. Patients were randomized by a closed envelope method generated by the research coordinator in a 1:1 ratio to either oral vancomycin (250 mg every 6 h for 10–14 days) or a FMT-FURM via a nasojejunal tube, superior endoscopy, or colonoscopy.

The decision on the modality of delivery of FMT-FURM was made in coordination with the attending physician and gastroenterology team and considered the patient's characteristics and planned procedures.

Allocation only occurred once the study investigators received an order to administer treatment from the coordinator. A patient ID was assigned to each participant and samples collected were identified with this ID (no details of the study group were included in the ID). Samples were sent to the laboratory.

Randomized patients received treatment during the study period according to the intervention they were allocated. Laboratory investigators were blinded to treatment allocation.

### Definitions

CDI resolution was considered when at least 2 of the following criteria were met: a) a reduction on the Bristol scale of at least 2 points, b) a reduction of at least 50% in the number of bowel movements during the first 72 h after the FMT-FURM, c) absence of fever, and d) resolution of abdominal pain. For patients with initial leukocytosis, normalization of values was considered. Treatment was considered to have failed when ≥3 of the resolution criteria were not met within 72 h. Fecal microbiota re-transplantation was performed by administering a second dose of FMT-FURM within 72 h of the first FMT-FURM.

### Selection of feces donors

Potential donors of either sex were over 18 years old, non-pregnant, with a body mass index of 20–25 kg/m^2^, with a normal total blood count and normal serum levels of liver enzymes, as well as a medical history without any of the following: consumption of proton-pump inhibitors, antibiotics, immunosuppressive medication, hospitalization, and diarrhea three months prior to donation. Additional exclusion criteria were: high-risk sexual behavior, a first-degree relative with diabetes mellitus, abdominal surgery, and any gastrointestinal disease or cancer.

Furthermore, potential donors were excluded if they tested positive for any of the following pathogens: hepatitis A virus, hepatitis B virus, hepatitis C virus, human immunodeficiency virus 1–2, *Trypanosoma cruzi*, *Brucella* sp., *Treponema pallidum*, cytomegalovirus, Epstein-Barr virus, parasites, enteropathogenic bacteria, and rotavirus. A potential donor with a positive test for any of these pathogens was excluded from the study. Feces donors were not requested to be on a specified diet before the donation.

### Preparation of pooled fecal microbiota samples and storage

From each donor, three stool samples were collected within 2 weeks after the evaluation of the donor. Feces from all donors were pooled, mixed, resuspended in 0.9% saline, and filtered to remove particles > 330 μm. Glycerol, at a final concentration of 15% (v/v), was added as a bacterial cryoprotectant before the mixture was stored in 45-mL aliquots at -80°C. Before use, a 45-ml -aliquot was thawed within 60 minutes by immersion in water at 30°C and administrated to the patient. A 45-ml aliquot was considered a doses.

### Patient variables, follow-up, and re-transplantation

Patient variables that were monitored after CDI diagnosis and enrollment included: frequency of bowel movements, stool consistency, gastrointestinal symptoms, fever, and the use of antibiotics due to other pathologies. Additional variables that were registered were the use of proton pump inhibitors, a previous stay at the intensive care unit, prior orotracheal intubation, immunosuppression or renal replacement therapy, 30-day mortality, and attributable mortality. At baseline and 24 h, 48 h, and 72 h after treatment, ATLAS, APACHE II, and SOFA scales were determined. Patients in either arm without clinical improvement at 72 h after the first treatment received a second treatment which was a FMT-FURM in all cases. Data monitoring and calculations were carried with the moment of second treatment as the reference time point. Follow-up of patients comprised from the beginning of the study in February 2015 to February 2016. The study was ended when the minimum number of patients for each arm was completed.

### Culture, genotyping, and drug susceptibility of *C*. *difficile*

Besides clinical trial, feces samples were cultured to recover and to characterize isolates. Feces samples from patients, collected on days 0 (pre-treatment), 3, and 7 after treatment, received a 3-hour ethanol shock before inoculation onto cycloserine-cefoxitin fructose agar (CCFA). Plates were incubated in an anaerobic chamber at 37°C for 48 h. Colonies were identified with both a matrix-assisted laser desorption ionization time-of-flight (MALDI-TOF) mass spectrometer (Brüker Daltonik) and triose phosphate isomerase (*tpi*) genotyping by polymerase chain reaction (PCR) [11]. Hereto, five *C*. *difficile* colonies were resuspended in 200 μL 100 mM Tris-HCl, lysed by adding 150 μg lysozyme, and incubated at 37°C overnight. Genomic DNA was extracted with a standard phenol-chloroform-isoamyl alcohol protocol. The genes *tcdA*, *tcdB*, *cdtA*, and *cdtB* were amplified using a multiplex PCR as described [12]. For *tcdC* internal in-frame deletion analysis, PCR was performed as described by Persson *et al*. [12].

The MICs of ciprofloxacin, moxifloxacin, erythromycin, clindamycin, vancomycin, metronidazole, linezolid, fidaxomicin, and tetracycline against *C*. *difficile* isolates were determined by the agar dilution method [13]. Briefly, bacteria were cultured anaerobically in Schaedler's anaerobic broth (Oxoid, Basingstoke, UK) at 37°C for 48 h, and were multipoint inoculated (10^4^ colony-forming units (CFU)/spot) onto Wilkins–Chalgren agar (Oxoid) containing 2-fold serial dilutions of each antibiotic (0.03–512 mg/L).

Previously reported breakpoints were considered for ciprofloxacin [14], linezolid [15], and rifampicin [16]. Vancomycin breakpoints were those from the EUCAST Clinical Breakpoint Tables v. 6.0, 2016, and the breakpoints of the other antimicrobials were obtained from the clinical CLSI guideline M100-S26 [17].

### Microbiota analysis

Stool samples obtained at days 0, 3 and 7 were analyzed using 16S rRNA sequencing. The sample from the donors’ pool (one sample only) was also analyzed as a reference for changes in the FMT-FURM group.

The semi-conserved V4 region of the16S rRNA gene was amplified with primers recommended by the Earth Microbiome Project [18]. The PCR amplicons were sequenced on a MiSeq instrument (Illumina) at Molecular Research LP (Shallowater, Texas, USA) according to the manufacturer’s instructions. Full.fasta and full.qual files provided by MRDNA (these files contain joined reads ranging from >300 to <570 bp) were used. Demultiplexing (*i*.*e*., assignment of 16S rRNA reads to samples) and quality filtering were performed using default parameters in the split_libraries_fastq.py script in QIIME 1.8. Operational taxonomic units (OTUs) were defined using an open-reference algorithm [19] that not necessarily discarded sequences that were not present in the reference sequence file GreenGenes 13.5 [20].

Several diversity analyses were performed using both the OTU table, generated in the previous step and the core_diversity_analyses.py script in QIIME, with minor modifications. More importantly, we removed very low abundant OTUs (*i*.*e*., OTUs with <0.005% sequences) to increase the sensitivity of detecting true phyla [21]. Also, the original OTU table was filtered to allow independent analysis of both FMT-FURM and vancomycin groups. All 16S data and associated metadata were uploaded into the Sequence Read Archive at the NCBI (BioProject ID: PRJNA414451).

### Statistical analysis

Baseline clinical and demographic characteristics were analyzed using χ^2^ test for categorical data and Students´ *t*-test or Mann-Whitney test depending upon the distribution of the data.

Bacterial abundance was expressed as a relative proportion (*i*.*e*., percentage) and analyzed using linear discriminative analysis (LDA) effect size (LEfSe) [22] within each treatment group. The default LDA score of 2 and *P* value of 0.05 was used for the Kruskal-Wallis test. Taxa that were identified by LEfSe to be associated with a given time point were further compared using pairwise Mann-Whitney with Bonferroni correction using Paleontological Statistics Software (PAST) [23]. Alpha diversity metrics included the number of OTUs (species richness), the PD whole tree and Shannon diversity indexes, and the Chao1 metric. The Kruskal-Wallis was used in a PAST environment to compare the groups[24]. Beta diversity was analyzed using both unweighted[25] and weighted[26] UniFrac distances in PAST [23].

A principal coordinate analysis (PCoA) was used to analyze the clustering of microbial communities using the Unifrac distances. The Adonis and ANOSIM tests were used to explore significant clustering of communities according to a time point in QIIME.

## Results

### Donor and study population

Twenty-one donors were interviewed, but only 3 (mean age, 23.7 years) fulfilled all inclusion criteria and were enrolled (Table 1). In total, 881.62 g of feces were collected. The final suspension had a concentration of 0.19 g/mL.

**Table 1:** Demographic and laboratory characteristics of feces donors.

	Donor 1	Donor 2	Donor 3	Average
Gender	Male	Female	Female	
Age (years)	20	27	24	23.67
Body mass index (kg/m^2^)	24.09	24.12	20.26	22.82
Hemoglobin (g/dL)	17.8	15	13.8	15.53
Leukocytes (10^3^/μL)	7.7	6.9	4.6	6.40
Platelets (10^3^/μL)	266	375	252	297
Glucose (mg/dL)	83	87	88	86
Creatinine (mg/dL)	0.8	0.7	0.6	0.7
Albumin (g/dL)	4.5	4.0	4.5	4.33
Alanine amino transferase (UI/L)	19	17	20	18.67
Aspartate amino transferase (UI/L)	19	10	11	13.33
Total bilirubin (mg/dL)	1.7	0.7	0.9	1.1
Cholesterol (mg/dL)	171	190	74	145
Triglycerides (mg/dL)	72	46	57	58.33

**Fig 1:**
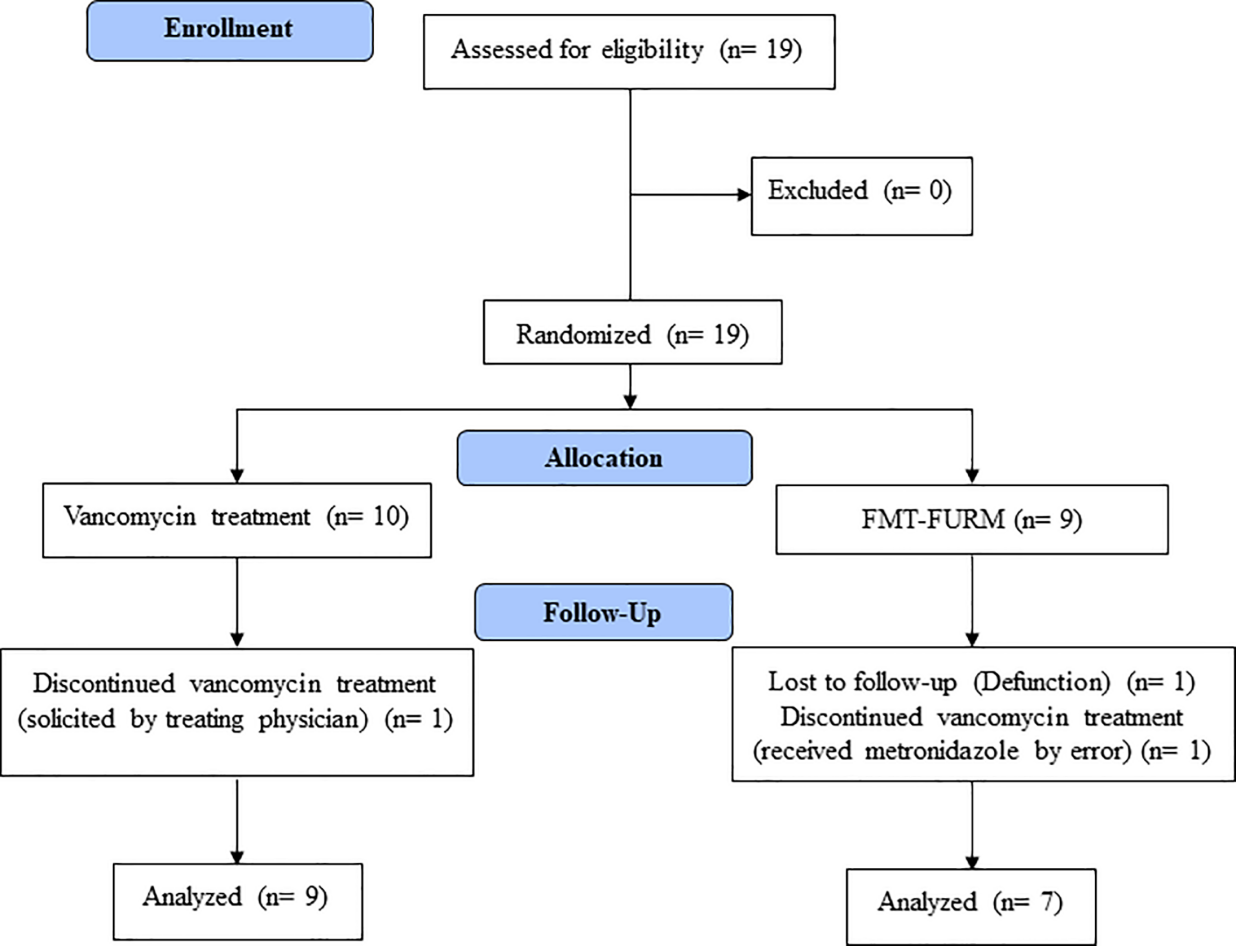
Flowchart of the study design.

We included 19 patients; 10 in the vancomycin arm and 9 in the FMT-FURM arm. One of the patients in the vancomycin arm was removed from the protocol at the request of the treating physician. Two patients in the FMT-FURM arm were eliminated from the study; one died during the study because of an unrelated cause and the other one received oral metronidazole due to human error (Fig 1). Demographic and main clinical characteristics of the patients are presented in Table 2: nine patients in the vancomycin group (mean age: 46.7 ± 15.8 years, range: 19–70 years; female, n = 3) and 7 in the FMT-FURM group (mean age: 39.7 ± 24.8 years, range: 18–91 years; female, n = 3).

**Table 2:** Demographic and main clinical characteristics of patients in vancomycin and FMT-FURM groups.

Reg Patient	Age (years)	Gender	LOS after CID (days)	Comorbidities	Systemic antibiotic	Outcome	30 day-mortality	CDI attributable mortality
Vancomycin group								
1	70	F	8	CKD, DM2	No	Resolved	No	No
2	53	M	19	PO abdominal abscess	Yes	Resolved	No	No
4	19	F	6	SLE, CKD	Yes	Resolved	yes	No
11	58	M	10	Hemorrhagic CVD	No	Resolved	No	No
12	60	M	6	Hemorrhagic CVD	Yes	Resolved	Yes	No
13	34	F	4	PO hysterectomy	No	Resolved	No	No
14	45	M	5	Pulmonary TB	Yes	Fail	Yes	Yes
15	33	M	4	Complicated pleural effusion	Yes	Resolved	Yes	No
8	48	M	6	Child B liver disease	No	Resolved	No	No
FMT-FURM								
3	39	M	29	Abscessed squamous cell cancer	Yes	Fail	Yes	Yes
5	18	M	36	CKD, HD	Yes	Fail	No	No
6	18	F	9	SLE	Yes	Resolved	No	No
7	30	F	9	CKD, HD	Yes	Resolved	No	No
9	91	F	6	Stevens-Johnson Syndrome	No	Resolved	No	No
10	39	M	14	Thrombocytosis’ essential	No	Resolved	No	No
16	43	M	7	Meningeal TB	Yes	Resolved	Yes	No

Ca, carcinoma; CKD, chronic kidney disease; CVD, cerebrovascular disease; DM2, type 2 diabetes; FMT, transplantation of fecal microbiota; HD, hemodialysis; LOS, length of stay; PO, postoperative; SLE, systemic lupus erythematosus; TB, tuberculosis; V, vancomycin.

FMT-FURM was administered by upper endoscopy in all but 2 cases, who received the FMT by a nasojejunal tube that had already been installed (n = 1) or by colonoscopy in a case with anatomical abnormalities due to head and neck neoplasia (n = 1).

### Clinical data

Regarding the clinical characteristics of the patients, leukocyte counts were higher in the vancomycin group than in the FMT-FURM group (*P* = 0.04). All other variables regarding clinical and laboratory data previous to CDI, at diagnosis or outcome, did not show statistical differences between the 2 groups (Table 3). It is important to underline that this study is far underpowered and should be considered as a pilot study.

**Table 3:** Clinical and demographic data of patients in the vancomycin and FMT-FURM groups.

	Vancomycin (n = 9)	FMT-FURM (n = 7)	P
Male n (%)	6 (66.7)	4 (57.1)	0.69
Mean age (years ±SD)	46.7 ± 15.8	39.7 ± 24.8	0.50
**Previous to CDI**			
Endotracheal intubation, n (%)	1 (11.1)	4 (57.1)	0.10
ICU stay, n (%)	1 (11.1)	2 (28.6)	0.55
Hemodialysis or peritoneal dialysis, n (%)	1 (11.1)	2 (28.6)	0.55
**At diagnosis**			
Charlson score	1.6 ± 1.7	2.4 ± 2.6	0.43
ATLAS score	6.0 ± 1.9	4.9 ± 1.7	0.22
APACHE II	11.00 ± 5.52	11.71 ± 6.29	0.81
SOFA score	11.00 ± 5.52	10.00 ± 4.36	0.70
Concomitant pharmacotherapy, n (%)	5 (55.6)	5 (71.4)	0.63
Immunosuppression, n (%)	2 (22.2)	2 (28.6)	0.77
Proton pump inhibitors use, n (%)	5 (55.6)	6 (85.7)	0.30
Temperature ≥38°C, n (%)	4 (44.4)	5 (71.4)	0.35
Abdominal pain, n (%)	6 (66.7)	5 (71.4)	1.00
Leukocytes (10^3^/μL) (mean ±SD)	23.3 ± 12.0	12.9 ±6.9	0.04
Albumin (mean ± SD)	2.1 ± 0.6	2.3 ±0.8	0.55
**Outcome**			
30-day mortality, n (%)	4 (44.4)	2 (28.6)	0.63
Attributable mortality, n (%)	1 (11.1)	1 (14.3)	0.84
Treatment failure, n (%)	1 (11.1)	2/7 (28.5)	0.06
Change in bowel movements/day (mean ±SD)	22.1 ± 88.2	12.6 ± 37.7	0.79
Change in ATLAS score (mean ±SD)	15.9 ± 34.8	26.4 ±31.9	0.54
Change in SOFA score (mean ±SD)	12.51 ± 31.2	-0.4 ± 50	0.53
Change in APACHE (mean ±SD)	12.5 ± 31.2	7.0 ± 50.4	0.79

ICU: Intensive care, APACHE II Acute physiology and chronic health evaluation, SOFA sequential organ failure assessment.

**Fig 2:**
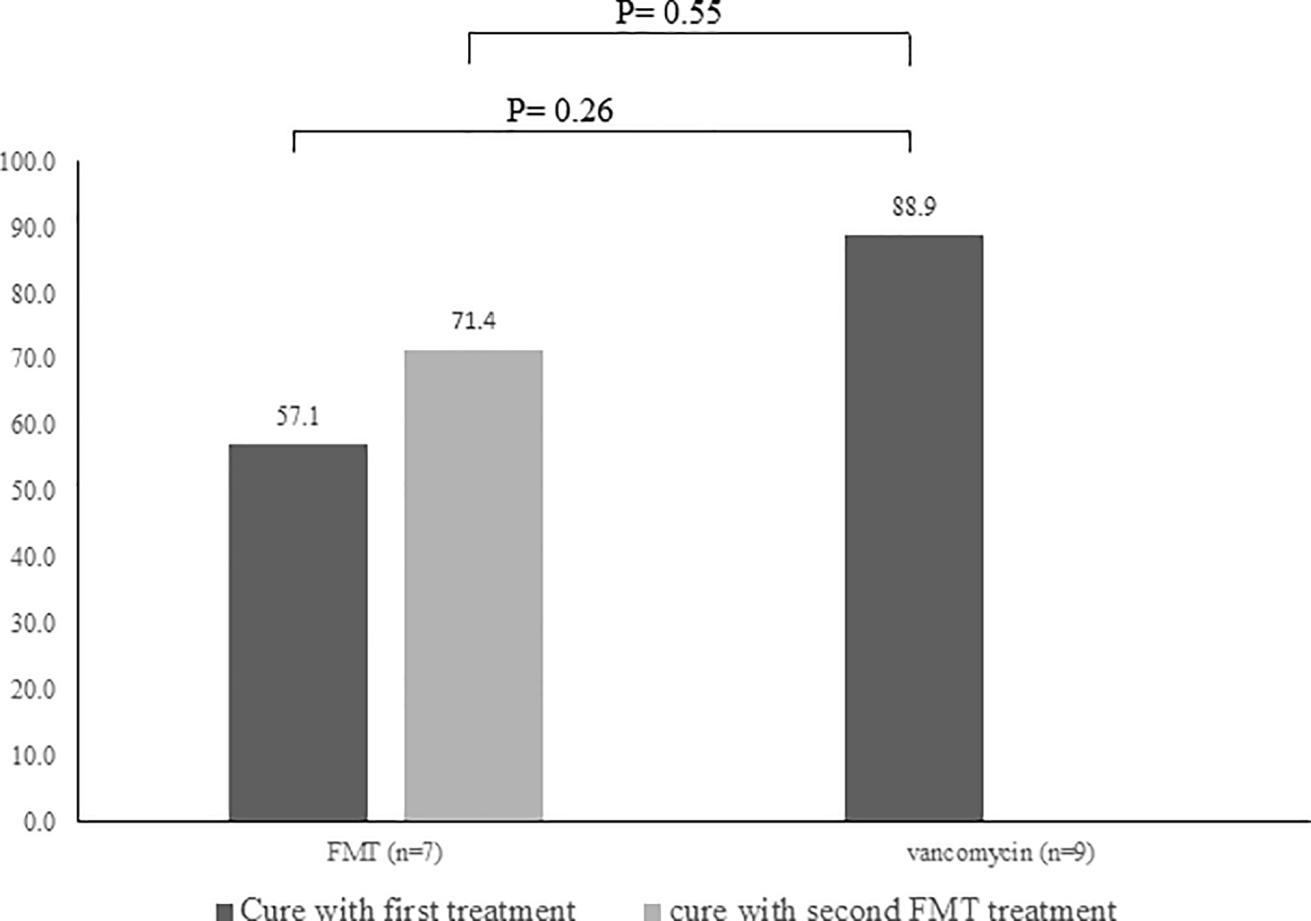
Resolution rates (%) of initial *C*. *difficile* infection in patients treated with vancomycin and fecal microbiota transplantation (FMT).

Symptoms resolved in 8/9 patients (88.9%) in the vancomycin group, in 4/7 (57.1%) patients the FMT-FURM group after the first FMT-FURM (*P* = 0.26), and in 5/7 (71.4%) patients after the second FMT-FURM treatment (*P* = 0.55) (Fig 2).

### Genotypes

We recovered 12 clinical isolates, 7 from day-0 and 5 from day-3 samples (Table 4). Most isolates from day 0, 6/7 (85.7%), carried genes encoding enterotoxin, cytotoxin, and the binary toxins (*tcdB*, *tcdA*, *cdtA*, and *cdtB*), and these strains carried the putative negative regulatory gene *tcdC*, with an 18-bp deletion (NAP1/027). In specimens from day 3, 4/5 (80%) were NAP1/027.

**Table 4:** Distribution of cultures, genotypes, and MICs (μg/mL) among fecal specimens collected on days 0 and 3.

Group	Reg. patient	Day	Culture	Genotype	CIP^a^		MOX*		ERY*		CLI*		VAN^b^		MET*		LIN^c^		FDX*		RIF^d^			TET*	
					MIC	INT	MIC	INT	MIC	INT	MIC	INT	MIC	INT	MIC	INT	MIC	INT	MIC	INT		MIC	INT	MIC	INT
FMT	10	0	pos	*tcdB*, *tcdA +*	16	R	16	R	2	S	2	S	1	S	0.5	S	1	S	0.06	S		0.002	S	0.03	S
FMT	10	3	pos	*tcdB*, *tcdA +*	16	R	32	R	4	S	4	S	1	S	0.5	S	1	S	0.06	S		0.002	S	0.06	S
VAN	15	0	pos	NAP1/027	>128	R	64	R	>128	R	>128	R	8	R	2	S	4	S	0.015	S		>128	R	0.03	S
VAN	15	3	pos	NAP1/027	>128	R	64	R	>128	R	>128	R	8	R	2	S	1	S	0.008	S		>128	R	0.03	S
FMT	5	0	pos	NAP1/027	>128	R	64	R	>128	R	>128	R	4	R	2	S	4	S	0.015	S		>128	R	0.03	S
FMT	5	3	pos	NAP1/027	64	R	64	R	>128	R	>128	R	4	R	2	S	4	S	0.015	S		>128	R	0.03	S
VAN	3	0, 3	pos	NAP1/027	>128	R	64	R	>128	R	>128	R	4	R	2	S	4	S	0.015	S		>128	R	0.03	S
VAN	14	0, 3	pos	NAP1/027	64	R	64	R	>128	R	>128	R	4	R	2	S	1	S	0.015	S		>128	R	0.03	S
FMT	2	0	pos	NAP1/027	>128	R	64	R	>128	R	>128	R	4	R	2	S	4	S	0.015	S		>128	R	0.03	S
VAN	7	0	pos	NAP1/027	64	R	64	R	>128	R	>128	R	4	R	2	S	1	S	0.015	S		>128	R	0.03	S
VAN	12	0	pos	NAP1/027	>128	R	64	R	2	S	>128	R	4	R	2	S	4	S	0.015	S		>128	R	0.03	S
VAN	11	0	pos	NAP1/027	>128	R	64	R	>128	R	>128	R	4	R	2	S	4	S	0.015	S		>128	R	0.03	S
FMT, VAN	2,7, 11, 12	3	neg	NA	-		-		-		-		-		-		-		-			-		-	
FMT, VAN	1, 4, 6, 8, 9, 13	0, 3	neg	NA	-		-		-		-		-		-		-		-			-		-	

NAP1/027: *tcdB*, *tcdA*, *cdtA*, *cdtB*, 18bp deletion +. CIP: ciprofloxacin; MOX; moxifloxacin; ERY: erythromycin; CLI: clindamycin; VAN: vancomycin; MET: metronidazole; LIN: linezolid; FDY: fidaxomicin; TET: tetracycline. NA: Not Applicable. INT: Interpretation.

^a^Büchler *et al*. 2014

^b^EUCAST

^c^Goldstein *et al*. 2013; O’Connor *et al*. 2008

*CLSI, 2016

In five cases, feces samples from both day 0 and 3 yielded positive cultures, with 4 of them being NAP1/027. In four cases, only the day-0 (but not the day-3) sample produced a positive culture which was NAP1/027 in all cases. In 7 other cases, samples from both days yielded a negative culture result (Table 4).

All isolates were resistant to ciprofloxacin and moxifloxacin but susceptible to metronidazole, linezolid, fidaxomicin, and tetracycline. Except for 1 cases, (day-0 and day-3 samples from patient 10, FMT group), drug susceptibility did not differ between isolates of days 0 and 3.

A patient that failed to recover from infection in the vancomycin group, (patient 14) had a *C*. *difficile* isolate that was vancomycin-resistant (MIC = 4).

### 16S rRNA analysis

For 16S rRNA analysis, at least 2 samples (days 0 and 3) were included from 14 patients; from 8 of them, a third sample was obtained (day 7), so that a total of 36 specimens were evaluated. Two patients were not included in the analysis as there was only 1 sample/case.

A total of 8,369,804 raw16S rRNA sequences were generated from 37 samples (36 case samples + 1 samples from the donor pool) with the MiSeq instrument. After demultiplexing and quality filtering, 7,169,921 good-quality 16S rRNA sequences (lowest: 63,458, highest: 294,094) were available for OTU picking, taxonomic assignments, and further diversity analysis. The removal of low abundant OTUs with an open reference algorithm [19] yielded a total of 803 OTUs. Diversity analyses were performed using a rarefaction of 50,000 (lowest number of sequences per sample).

### Metagenomic analysis across groups

**Fig 3:**
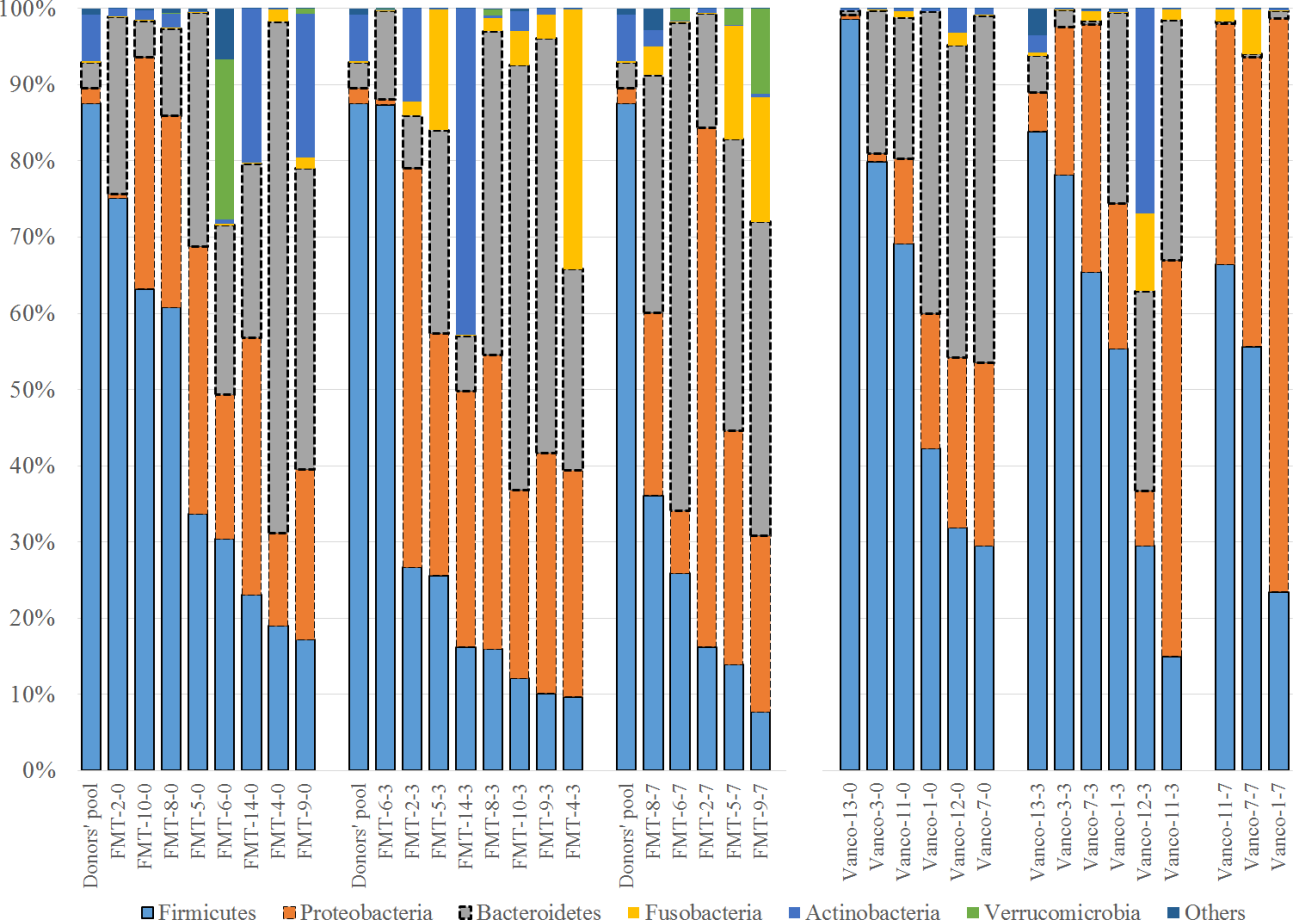
Bar charts showing the relative abundance of OTUs at the phylum level. Distribution of bacterial phyla in the fecal microbiota of transplantation (FMT) group and the vancomycin group (Vanco). The label of each sample (e.g., FMT-2-0) denotes Treatment (FMT or Vancomycin), the assigned number of each patient, and days on treatment (0 = baseline, 3 = 3 days on treatment, 7 = 7 days on treatment). To facilitate comparison and visualization, the distribution of bacterial phyla of the donors’ pool is presented for the FMT group (Donors’ pool). Please note that the border of the three most abundant groups was highlighted for better visualization and the bars were organized based on the abundance of the highest abundant group (i.e., Firmicutes).

Firmicutes, Bacteroidetes, and Proteobacteria were the most abundant phyla, comprising ~90% of all bacteria in the samples. Interestingly, Firmicutes were by far the most abundant phylum in the donors’ pool (88%); by contrast, only 2/36 patient samples had a similar (87.4%) or higher (98.6%) relative abundance of Firmicutes. Besides, the donors’ pool had a partially low relative abundance of Proteobacteria as only four samples had low Proteobacteria relative abundance. The donor´s pool also had relatively few Bacteroidetes, as only six samples contained lower relative abundances of Bacteroidetes (Fig 3).

**Fig 4:**
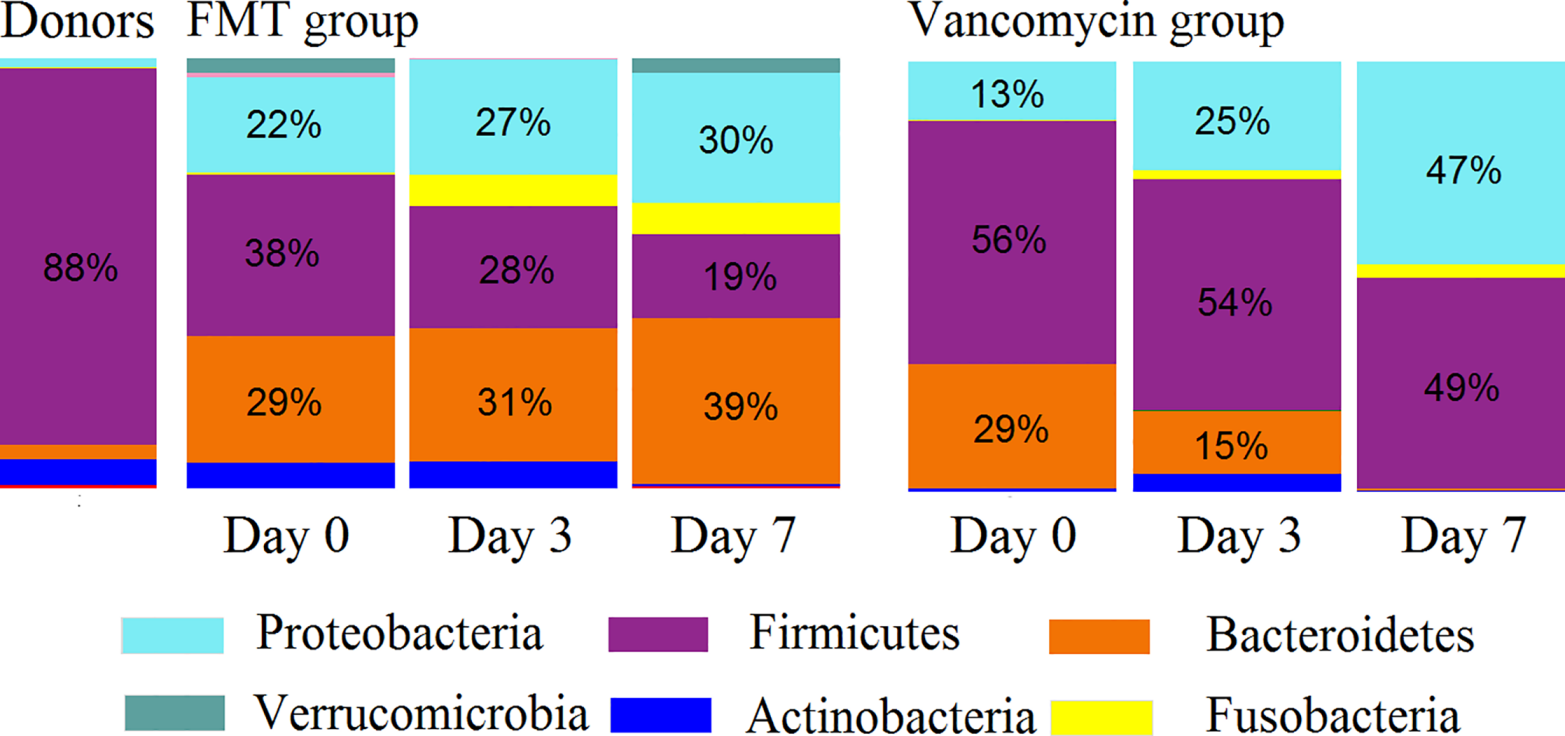
Average bacterial composition at the phylum level. FMT: fecal microbiota transplantation.

In the FMT-FURM group, the bacterial composition was dominated by Firmicutes, Bacteroidetes, and Proteobacteria at all-time points (day 0, 3 and 7) and the microbiota were remarkably stable over time (Fig 4).

**Fig 5:**
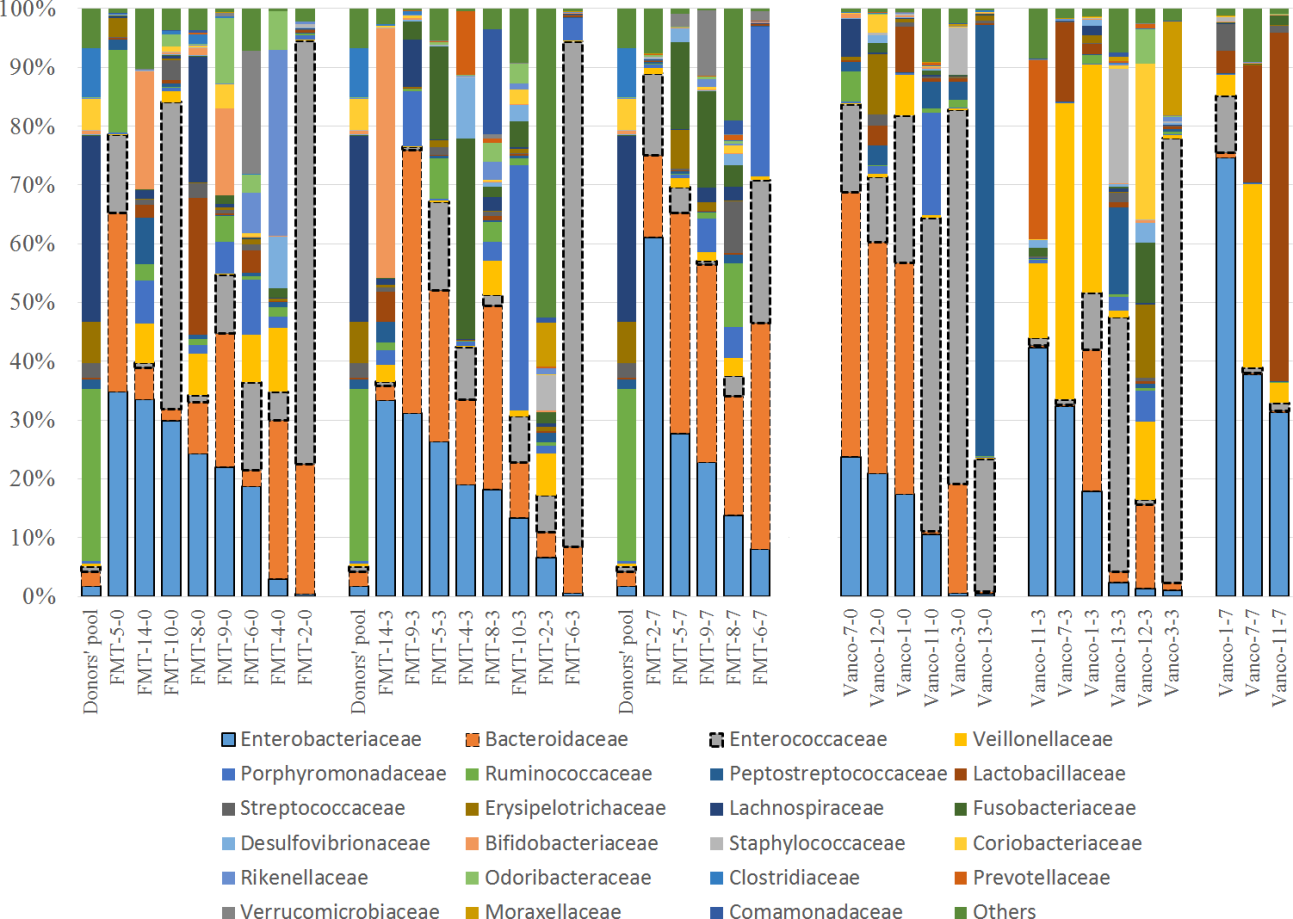
Bar charts showing the relative abundance of OTUs at the family level. Distribution of bacterial families in the fecal microbiota of transplantation (FMT) group and the vancomycin group (Vanco). The label of each sample (e.g., FMT-5-0) denotes Treatment (FMT or Vancomycin), the assigned number of each patient, and days on treatment (0 = baseline, 3 = 3 days on treatment, 7 = 7 days on treatment). To facilitate comparison and visualization, the distribution of bacterial families of the donors’ pool is presented for the FMT group (Donors’ pool). Please note that the border of the three most abundant groups was highlighted for better visualization and the bars were organized based on the abundance of the highest abundant group (i.e., Enterobacteriaceae).

Despite the predominance of Firmicutes in the donors’ pool (88%), the proportion of Firmicutes in the FMT-FURM group remained relatively small and, in fact, decreased over time. In this study, Firmicutes were mostly related to Lactobacillales and Clostridiales, bacterial orders mainly containing organisms known to be beneficial to gut homeostasis (Fig 5).

There was no statistically significant difference in Firmicutes or any other bacterial group within this important phylum among the three-time points.

The vancomycin group showed a very different pattern of the microbial composition when comparing to the FMT-FURM group over time (Fig 4). For example, Bacteroidetes decreased from an average of 29% (day 0) to 15% on day 3, and then near 0% on day 7; mostly *Bacteroides* spp. were affected. By contrast, Proteobacteria increased from as little as 13% (day 0) to 47% on day 7, and the increase was mostly due to members of Enterobacteriaceae (Fig 5). However, no taxon displayed significant differences among the three-time points. The lack of significant difference in microbiota composition among the three-time points was also observed individually in the three patients that had complete sample sets (Fig 4). In the vancomycin group, Firmicutes, mostly Clostridiales and Lactobacillales, remained relatively high (~50%) and stable over time (Fig 5).

### Diversity analysis

**Fig 6:**
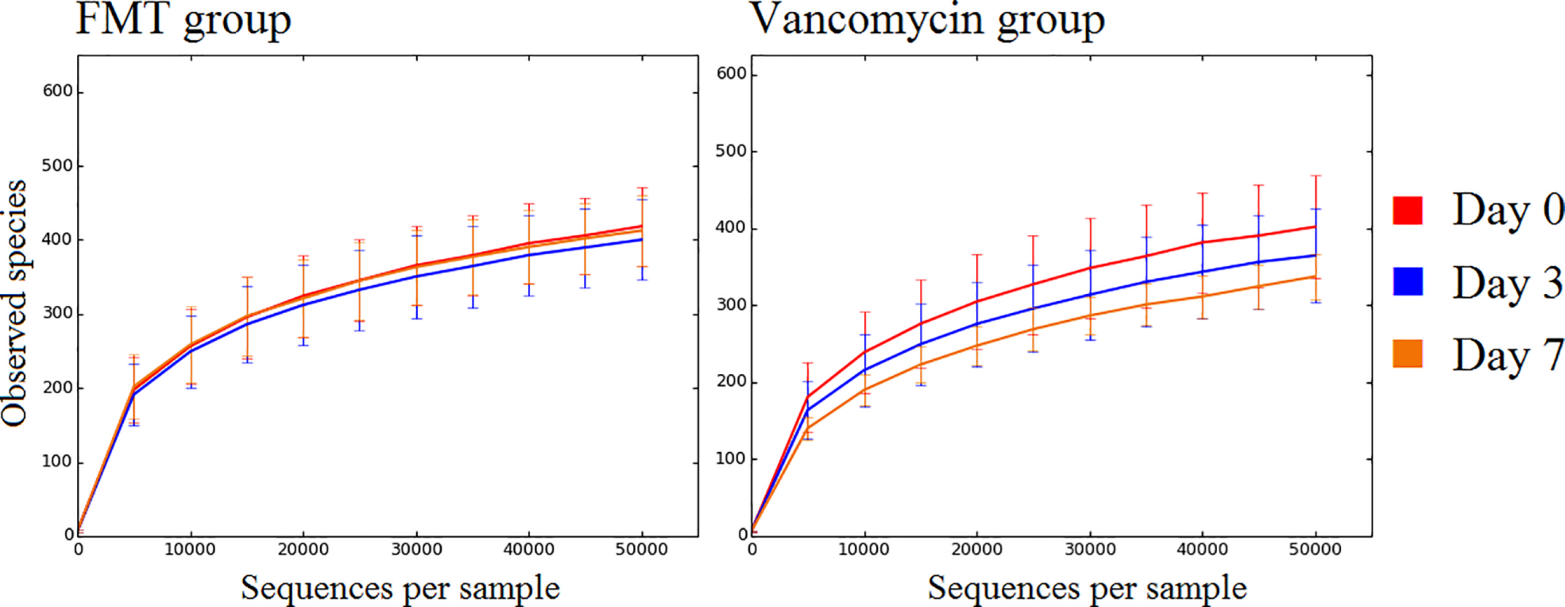
Number of observed species. The figure shows the number of observed species (*i*.*e*., operational taxonomic units at 97% similarity, y-axis) and the number of sequences per sample (x-axis) for the FMT group and Vancomycin group across collection days of fecal samples. Error bars represent standard deviations.

In the vancomycin group, a pattern in the number of species (i.e., OTUs at 97% similarity) over time was observed. On average, the first sample had the highest and the last sample the lowest numbers of OTUs (Fig 6).

**Fig 7:**
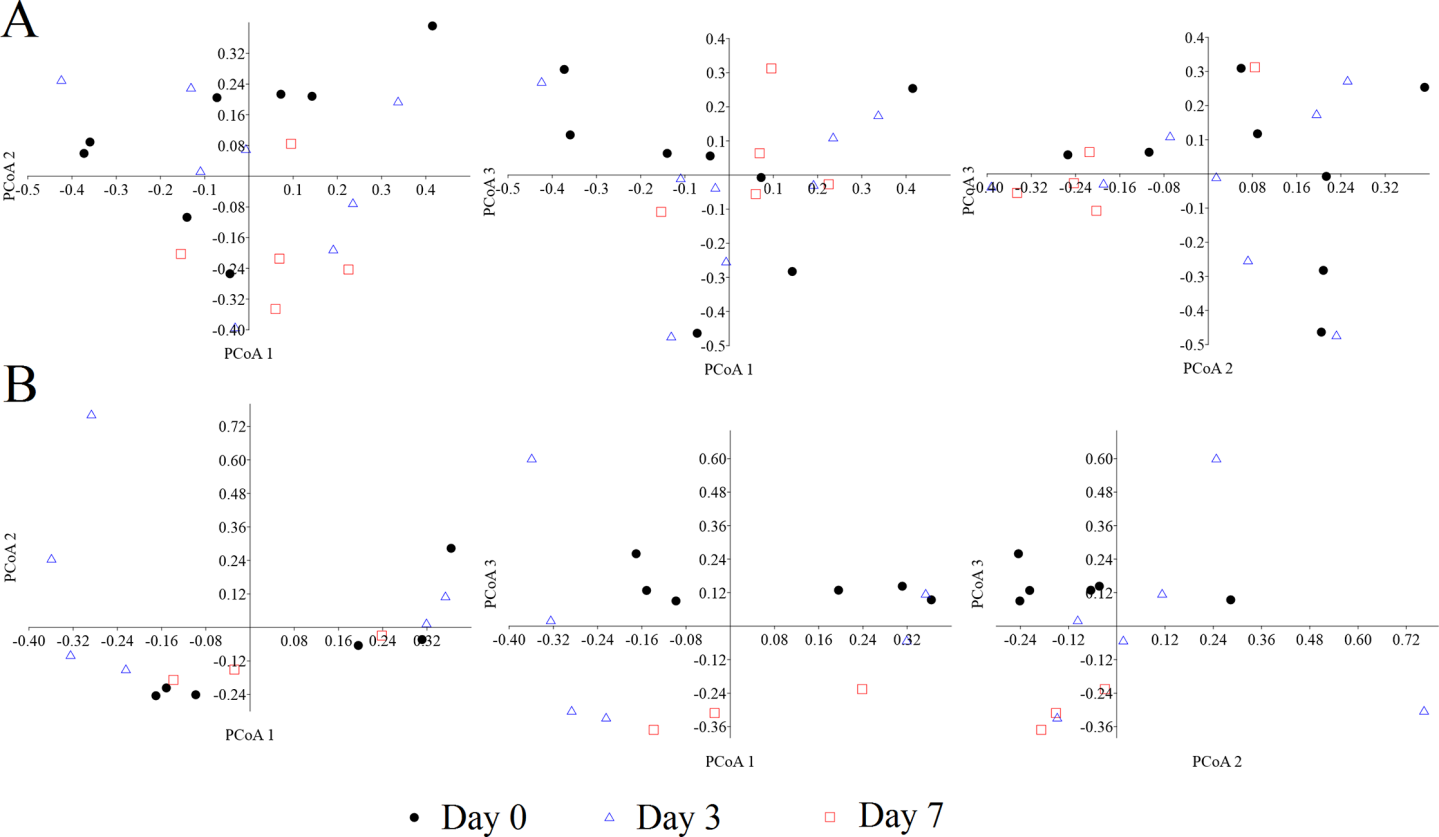
PCoA plots of weighted UniFrac distances for the FMT group and the vancomycin group. PCoA plots of weighted UniFrac distances for both groups across the days of the collection of fecal samples. There was no significant clustering of microbial communities according to time point (please note that the analysis of *unweighted* UniFrac distances did not reveal any significant clustering of microbial communities either).

However, this pattern did not reach statistical significance (Table 5). Despite the lack of statistical significance, all alpha diversity metrics tended to decrease over time in the vancomycin group; a pattern that was not observed in the FMT-FURM group. A PCoA of both weighted and unweighted UniFrac distances revealed no clear separation among the three-time points (Fig 7), suggesting that the microbial communities remained stable over time despite the differences in bacterial composition and diversity.

**Table 5:** Median (minimum-maximum) alpha diversity metrics in the FMT-FURM and vancomycin groups.

	FMT-FURM group				Vancomycin group			
	Day 0 (n = 8)	Day 3 (n = 8)	Day 7 (n = 5)	P *	Day 0 (n = 6)	Day 3 (n = 6)	Day 7 (n = 3)	P *
Number of species	404 (346–506)	400 (324–503)	391 (369–505)	0.80	408 (294–472)	357 (295–447)	328 (312–382)	0.46
Shannon	4.5 (3.2–5.7)	4.7 (2.5–5.7)	4.7 (3.8–5.9)	0.98	4.2 (2.5–5.7)	3.7 (2.9–5.1)	3.5 (3.5–4.1)	0.77
Chao1	500 (480–599)	464 (410–541)	506 (464–590)	0.08	516 (381–567)	445 (369–529)	452 (425–482)	0.17
PD Whole tree	54.1 (48.3–61.8)	53.5 (44.2–63.2)	51.5 (50.0–62.4)	0.97	53.6 (46.8–57.3)	50.6 (46.0–56.9)	48.9 (47.7–49.9)	0.35

* P values come from the non-parametric Kruskal-Wallis test

The FMT-FURM group showed a very a similar number of species (Fig 6) and other alpha diversity metrics, except for Chao1 metrics (Table 5), among all three-time points (days 0, 3, and 7). A PCoA of both weighted and unweighted UniFrac distances revealed no separation among the 3-time points (*P* > 0.1 in both Adonis and ANOSIM tests) (Fig 7), suggesting that the microbial communities were not significantly different over time.

## Discussion

In this exploratory study, we aimed to evaluate the impact of FMT-FURM as first-line therapy of *C*. *difficile* infection in intestinal microbiome.

Most FMTs are performed with fresh feces from a related donor, but it has been proven that frozen feces can be used for FMT [1]. Feces specimens should be frozen with glycerol to ensure the viability of the microbiota. Freezing of feces allows reducing the time needed for transplant to be ready and reduces the risk of involving a donor that meets the minimum requirements and the transmission of infectious diseases through FMT. In our study, we used frozen feces to reduce the risk of potential FMT-related infections and to increase the availability of the FMT for critically ill patients.

We obtained from each donor three stool samples that were screened for the presence of virus, bacteria or parasite pathogens. The samples were mixed to generate a feces pool, with the intention of increasing the diversity of microbiota and potentially achieving a better clinical response. The donors were carefully selected to include an expected natural distribution of microbiota. We used a feces pool to minimize undetectable changes that may be present between donors.

During the study, no adverse effects attributable to FMT-FURM were observed in patients. However, long-term side effects cannot be discarded. In a multicenter long-term follow-up study of FMT for recurrent CDI, 77/94 eligible patients were contacted, and 4 patients had developed new disorders after FMT: peripheral neuropathy, Sjogren’s disease, idiopathic thrombocytopenic purpura, and rheumatoid arthritis [27]. On the other hand, in the same study, some patients reported improvement in pre-existing medical conditions, such as allergic sinusitis and arthritis. In this scenario, we intend to perform a long-term follow-up study in our patients.

Overall, our results revealed that the microbiome in the healthy donors´ pool was different from the microbial composition of all the patient samples, regardless of treatment, age, the number of transplants, etc.

The average age of the donors (23.7 years) was lower than the mean age of the patients (46.7 years in vancomycin group and 39.7 years in FMT group). It is known that the intestinal microbiome diversity differs according to age [28] and this phenomenon may have contributed to the apparent lack of FMT engraftment. We hypothesize that the use of FMT from donors of the same age as the patients may be more likely to graft.

We would like to point out some critical observations about the composition of the fecal microbiota in the vancomycin group as compared to the fecal microbiome of the FMT-FURM group. In all but one case of the vancomycin group, Bacteroidetes presence was less in the last sample (day 7) as compared to the first sample (day 0), which is in agreement with previously published studies that have evaluated the impact of vancomycin on the gut microbiota. We would like to highlight that there was no decrease in Bacteroidetes in the FMT-FURM group (considering they were receiving antibiotics because of other causes).

The fact that the recipient samples did not resemble the donors’ pool was also apparent in phyla other than Firmicutes. Despite the very low proportion of Bacteroidetes in the donors’ pool (only 3%), the levels of Bacteroidetes in the FMT-FURM group remained relatively high and increased by day 7. Also, the proportion of Proteobacteria (mostly Enterobacteriales) was, at all time points, greater in the FMT-FURM group than in the pooled donor sample. Furthermore, their Proteobacteria presence remained relatively stable over time. Again, these results are intriguing since the first sample is supposed to reflect a state of dysbiosis (due to CDI), yet this microbial consortium showed stability over time, a phenomenon probably supported by the FMT. Similar to Firmicutes, Bacteroidetes and Proteobacteria did not have significant differences in any bacterial group over time.

The microbial composition over time in the vancomycin group was different from the microbiome in the FMT-FURM group, which seems to reflect that oral antibiotics have a profound effect on the gut microbiota as has been reported before [29].

In this study, we observed an abundance of Firmicutes and Bacteroidetes on Day 3 of the vancomycin treatment group. These bacterial groups were expected to be highly altered in response to vancomycin therapy. Thus, the small perturbation observed in this community may be indicative of less damage to the microbiome and may have contributed to efficacy in this therapy group.

Apart from the fact that FMT-FURM was performed as an initial treatment for CDI, there are two additional interesting points that distinguish our study from other standard FMT treatments. Firstly, FMT is traditionally performed in an outpatient setting in recurrent cases (often in patients with ≥ 3 recurrences) who have received multiple treatments for CDI and are not currently receiving other antimicrobials. These factors profoundly influence the composition of the gut microbiome. Patients in the current study, however, were hospitalized at the time of treatment and, even though they had not received treatment for CDI, they were currently receiving or had recently received other antimicrobials for the treatment of infectious diseases other than CDI. The second interesting factor is that, as mentioned above, traditional FMT is performed in a stable and controlled environment and is preceded by enteral lavage, however, this was not performed in our study population. Given that the baseline and modifying factors are so different between the traditional FMT patients and our population, treatment response rates and microbiome changes should be carefully evaluated. We speculate that the exposure to antibiotics before and/or after FMT-FURM treatment negatively influenced the resolution of CDI even after re-transplantation.

It has been widely described the use of more than one infusion of FMT in the failure of response with up to four times for recurrent or unresolved symptoms. An efficacy of 70–75% has been observed with a single infusion, and this value increased to 85–90% when patients were given multiple FMT infusions [30]. In our study, the value observed was 71.4%, when a second infusion was administrated as needed. The lower value observed in our study may be due to the concomitant use of antibiotics. We would like to emphasize that this is a pilot study and we cannot conclude about therapeutic efficacy.

This study has several limitations a) the sample size was small. Thus the potential therapeutic benefits may not be interpreted such because of larger clinical studies, with a significant statistical power, need to be done, and results may show no therapeutic value. b) the programmed fecal samples for the microbiological examination were not obtained for all patients. c) the primary outcome in patients was diverse, and we could not use blinding to evaluators for assessing primary outcome. In this study, we did not intend to define the effect of delivery method: the treating physician decided the FMT-FURM route of administration in a way that would not affect the patient's care so that we do not obtain conclusions about the delivery method of the microbiota.

In conclusion, FMT for initial CDI is associated with specific bacterial communities that do not resemble the donors’ sample. To our knowledge, this is the first study to investigate the effect on fecal microbiota of FMT as a first-line treatment for CDI.

## Supporting information

S1 File Protocol in Spanish. Original document protocol (Spanish).
(DOCX)

S2 File Protocol in English. Translation of document protocol (English).
(DOCX)

S3 File Checklist. Information included in a randomized trial.
(DOC)

S4 File Strains information. Information of typing and strains susceptibility.
(XLSX)
