# Supplementary material for: Randomized clinical trial to evaluate the effect of fecal microbiota transplant for initial Clostridium difficile infection in intestinal microbiome
Source: PLoS One. 2017 Dec 20;12(12):e0189768. doi: 10.1371/journal.pone.0189768 (PMC5738078; doi:10.1371/journal.pone.0189768)
Supplement: S1 File — Original document protocol (Spanish). (DOCX) [file pone.0189768.s001.docx]

**“tratamiento inicial con trasplante de microbiota fecal en pacientes con infección por *Clostridium difficile”***

1. **Marco teórico**

La infección por *Clostridium difficile* (ICD) es un reto en la infecciones intrahospitalarias, y se ha incrementado en la última década e incluso ha superado la infección por *Staphylococcus aureus* meticilino resistente (MRSA) como la principal causa de infección intrahospitalaria. Esta infección es la causa más común de diarrea intrahospitalaria.

Además, se ha reportado la emergencia de una cepa de *C. difficile* resistente a quinolonas, la cual produce 16 veces más toxina A y 23 veces más toxina B que otras cepas de *C. difficile*.

El uso de antibióticos aumenta el riesgo de ICD 8 a 10 veces más por el siguiente mes y 3 veces por el segundo mes; existen múltiples asociaciones con el consumo de ampicilina, clindamicina y cefalosporinas de tercera generación, aunque el uso de cualquier antibiótico, (inclusive metronidazol) se ha asociado al desarrollo de ICD. El uso de múltiples antibióticos y más de 10 días con se ha asociado con el aumento de riesgo al desarrollo de ICD. Sin embargo la exposición a antibióticos no es necesaria para la adquisición de la enfermedad, en estos casos la enfermedad se asocia al contacto estrecho con pacientes con ICD. Se ha reportado que el 25% de los casos son en casas de asistencia a pacientes.

Otros factores asociados al desarrollo de ICD son el uso de antineoplásicos, los cuales alteran la microbiota fecal y son capaces de inducir arresto mitótico en células epiteliales, causando necrosis y descamación de la mucosa.

La edad es otro factor de riesgo asociado al desarrollo de ICD, con un aumento de riesgo de >10 veces para los pacientes de 60 a 90 años de edad. Se ha reportado que el 90 % de las muertes son en personas mayores de 65 años. Otros factores asociados a la ICD son el uso de enemas, estimulantes gastrointestinales y alimentación enteral postpilórica, esta última aumenta 11 veces el riesgo de desarrollar ICD.

Múltiples estudios han encontrado un riesgo elevado en usuarios de inhibidores de la bomba de protones, los cuales aumentan la habilidad de las esporas de *C. difficile* de convertirse en su forma vegetativa y sobrevivir en el lumen del tracto gastrointestinal; aun así el análisis multivariado no muestra diferencias significativas, en los estudios que en el análisis univariado mostro asociación directa; por lo que la prevención, detección y prevención de ICD no incluyen uso de inhibidores de bomba de protones.

La presentación clínica de la ICD varía desde infección asintomática, con el 20% de los casos, colitis con o sin pseudomembranas, hasta colitis fulminante y megacolon. La presencia de diarrea disentérica es poco común con solo el 5-10% de los casos, fiebre en el en 30 a 50% de los casos y solo 26% tienen sangre oculta en heces.

La leucocitosis es común en el 50 a 60% de los casos, hipoalbuminemia y elevación de azoados son altamente sugestivos de ICD. La hipoalbuminemia es resultado de de la pérdida de proteínas por enteropatía perdedora de proteínas. Los leucocitos fecales han sido encontrados en 28-40% de los casos.

El diagnóstico endoscópico se utiliza cuando no se puede hacer la determinación de toxinas A y B de *C. difficile* o cuando estas resultan negativas con una alta sospecha clínica. La endoscopia generalmente revela pseudomembranas y en 22% de los casos donde se observan macroscópicamente no se observan en histología.

El ensayo de neutralización y cultivo celular citotóxico es parcialmente aceptado como el estándar de oro diagnóstico es específico, aunque es costoso, no está estandarizado y está disponible solo en laboratorios de investigación.

El cultivo toxigénico se considera por algunos el método de elección. En el cultivo, las heces son cultivadas en un medio selectivo, posteriormente el organismo es probado para la producción de toxinas.

Por inmunoensayo ligado a enzima se determina la presencia de toxinas A y B, tiene una sensibilidad del 38% y un valor predictivo positivo de 50%. La falta de especificidad de la toxina A/B por EIA ha conducido a la búsqueda de métodos más precisos para diagnóstico. La detección de Glutamato deshidrogenasa (GDH) es prometedor, es un antígeno comúnmente expresado en altos niveles por cepas de *C. difficile*, sin embargo solo demuestra la presencia de *C. difficile*, no la presencia de cepas toxigénicas (el 20% de las cepas no lo son) ni la presencia de toxinas en heces, por lo que en caso de resultar GDH positvo requeriría un segundo examen y confirmar la presencia de toxinas.

El uso de reacción en cadena de la polimerasa (PCR) para detectar el gen productor de toxinas (tcdB), es rápido (aproximadamente 2 horas) con un límite de detección de 105/gr de heces, aunque el costo es 5 a 10 veces superior a la determinación de toxinas y su sensibilidad es de 91%. Sin embargo con esta prueba se detecta el gen productor de toxinas, más no la toxina; por lo que potencialmente se detectaría portadores asintomáticos. Además la prueba no puede ser utilizada para casos de recaída ya que el 56% de los pacientes tienen esta prueba positiva dentro de la semana 1 a 4 posterior a terminar tratamiento. Por lo anterior cuenta con valor predictivo positivo de 61%.

Actualmente las guías del Colegio Americano de Gastroenterología (ACG por sus siglas en inglés) recomiendan amplificación de ácidos nucleicos como PCR como el estándar de oro para diagnóstico de ICD. También sugieren que determinación de GDH puede ser usado como parte del algoritmo diagnóstico y confirmado con determinación de toxinas A/B por EIA. En caso de que éstas sean negativas entonces PCR.

El tratamiento es controversial hasta la fecha, porque no se puede establecer la eficacia de la terapéutica antibiótica, ningún antibiótico individual es claramente superior a otro. El uso de metronidazol es el antibiótico de preferencia por el desarrollo de enterococo resistente a vancomicina y el costo de vancomicina oral. Por lo que múltiples centros han sustituido las cápsulas de vancomicina por el uso de la formulación genérica intravenosa de vancomicina reconstituida en agua, utilizada como solución líquida vía enteral. Además de las diferencias por costo y disponibilidad del metronidazol y vancomicina no se han encontrado diferencias significativas entre el uso de ambos antibióticos. A pesar de respuestas similares con el uso de estos fármacos, existen diferencias farmacológicas que favorecen a la vancomicina, ya que el solo el 14% del total del metronidazol se elimina por heces, comparado con vancomicina ya que la absorción es prácticamente nula. A pesar de los niveles bajos de metronidazol *in vitro* se ha demostrado que bactericida rápido con solamente 8 veces la concentración mínima inhibitoria (CIM). Se ha demostrado que el 41% de los pacientes que reciben antibioticoterapia fallan a tratamiento en los primeros 14 días. Además se ha establecido papel para la rifampicina como tratamiento adyuvante con metronidazol.

La decisión terapéutica se basa en la gravedad del cuadro clínico. Según las recomendaciones del 2013 del ACG, clasifican el cuadro como leve a moderada, severa y severa complicada. El caso de cuadro leve o moderado se define como diarrea con alguna otra sintomatología que no cumpla con criterios de severidad se indica tratamiento con metronidazol vía oral (VO) y en caso de falta de mejoría en 5 a 7 días se consideraría cambio a vancomicina. En el caso de caso severo que incluye albúmina <3g/dl y leucocitosis >15,000cel/mm3 y/o dolor abdominal el tratamiento recomendado es vancomicina VO. El cuadro severo que incluye admisión en cuidados intensivos, hipotensión con o sin el uso de vasopresores, fiebre >38.5°C, íleo o distensión abdominal, alteración del estado mental, leucocitosis >35,000 cel/mm3, o leucopenia <2000 cel/mm3, lactato sérico >2.2mmol/L o falla orgánica; se recomienda el uso de vancomicina VO, simultáneo a vía rectal y metronidazol vía intravenosa. La justificación para el uso rectal de vancomicina es que para ser efectiva debe ser retenida y distribuida en el colon.

El papel del tratamiento quirúrgico se reserva para los casos que fallan al máximo manejo médico manifiestos como sepsis no resuelta, dilatación cecal mayor a 10cms (megacolon) o perforación intestinal. Con una incidencia reportada de 0.4-3.6% de los casos, de los cuales la mortalidad varia de 30 a 80%. El tratamiento óptimo en este punto es colectomía total ya que comparado con hemicolectomía la mortalidad aumenta con esta última de 11% a 100%.

Uno de los predictores más fuertes de mortalidad es el uso de vasopresores preoperatorio, que incrementa 4 veces el riesgo de muerte.

El trasplante de microbiota fecal se ha utilizado como una alternativa prometedora a la terapéutica sin antibióticos y se ha utilizado hasta el momento en pacientes quienes han sufrido múltiples recaídas o recurrencias. Usualmente de la materia fecal a trasplantar se obtiene de donador relacionado. Se ha utilizado en pacientes con ICD con una respuesta rápida y sin recurrencia en 86%, con pacientes libres de diarrea en meses o años. Los resultados de este tratamiento varían en las diferentes series con tipo de antibióticos usados previos a trasplante. Este tipo de terapéutica solo se ha explorado en casos refractarios o recurrentes. Una de las mayores limitantes es lo poco convencional de tratamiento además de ser estéticamente no placentero, a pesar de esto en estudios con los pacientes tratados con este método el 97% volvería a recibir el tratamiento y 53% lo hubiesen elegido como terapéutica inicial.

Un estudio en Holanda abierto, aleatorizado, controlado en 2013 (van Nood y col.) en pacientes con recaída de ICD comparó el tratamiento dividiendo a los pacientes en un protocolo de 3 brazos que comparaba vancomicina más trasplante de microbiota en el primer brazo, en el segundo vancomicina y lavado intestinal y en el tercer brazo tratamiento convencional con vancomicina. Los hallazgos fueron curación de 94%, 23 % y 31% respectivamente. Lo cual demuestra la eficacia terapéutica del trasplante de microbiota fecal.

Uno de los problemas que se pudiese enfrentar al realizar este tipo de trasplante radica en la obtención de muestras de heces fecales para trasplante. La posibilidad de un banco de muestras para trasplante puede ser una estrategia accesible y rápida. Dicha posibilidad quedó establecida cuando de 2004 a 2010 en el Hospital General del Sur de Estocolmo se realizaron trasplantes fecales de muestras obtenidas en 1994 de un solo donador.

**2. INVESTIGACIÓN CLÍNICA**

**2.1 Justificación**

La mortalidad en pacientes con infección por *Clostridium difficile* (ICD) es de 20% y es mayor en pacientes con infección severa. El trasplante de microbiota fecal tiene una tasa de curación de >90% en pacientes con IDC recurrente. El trasplante de microbiota fecal no se ha estudiado como primera línea de tratamiento en ICD.

**2.2Hipótesis**

**2.2.1Hipótesis verdadera**

El trasplante de microbiota fecal es superior a la antibióticoterapia como primera línea de tratamiento en pacientes con infección por *C. difficile*.

**2.2.2 Hipótesis nula**

El trasplante de microbiota fecal no es superior a la antibióticoterapia como primera línea de tratamiento en pacientes con infección por *C. difficile*.

**2.3Objetivos**

**2.3.1Objetivo primario**

Determinar la eficacia terapéutica del trasplante de microbiota fecal como primera línea de tratamiento en comparación con el uso de vancomicina vía entérica en pacientes con infección por *C. difficile.*

**2.4 Materiales y métodos**

**2.4.1Criterios de selección de pacientes y donadores**

Se incluirán pacientes mayores de 18 años, con diagnóstico de ICD por cualquiera de los siguientes métodos: detección de toxinas mediante inmunoensayo, prueba positiva por GeneXpert, cultivo positivo de heces para *C. difficile*, imagen colonoscópica sugerente de ICD. Se incluirán pacientes que acepten participar en el estudio mediante consentimiento informado firmado.

Se excluirán pacientes con megacolon tóxico, perforación intestinal sospechada o documentada, embarazo y la presencia concomitante de neoplasias de colon.

Se eliminarán los pacientes que decidan salir del estudio.

En cuanto a la selección de donadores, se les indagará sobre la historia de viajes, comportamiento sexual, cirugías previas, trasfusiones y otros factores de riesgo

Se incluirán sujetos sanos, donadores altruistas de sangre, que permitan acceder a información confidencial obtenida en Banco de Sangre del Hospital Universitario sobre sus resultados exámenes de laboratorio; información que los mismos donadores solicitarán al banco de sangre y nos la harán llegar al equipo de investigadores, los cuales trataremos la información de manera confidencial. Además los sujetos deberán tener índice de masa corporal de 20 a 25kg/m2, sin relación con los pacientes, con ausencia de historia de enfermedades autoinmunes, ausencia de diabetes o síndrome metabólico, que no hayan recibido antibióticos sistémicos en los últimos 3 meses, que no hayan recibido inhibidores de la bomba de protones en las 2 semanas previas a la recolección de las muestras, que ni hayan recibido inmunosupresores en los últimos 12 meses, que no tengan historia de enfermedades infecciosas transmisibles, que tengan ausencia de enfermedades infecciosas transmisibles, que no hayan presentado diarrea en los 3 meses previos a la recolección de la muestra, que no hayan sido hospitalizados en los últimos 3 meses y en el caso de mujeres, que no estén embarazadas.

Las muestras de heces obtenidas de los donadores deberán estar libres de los siguientes microorganismos: *C. difficile*, *Helicobacter pylori*, *Campylobacter jejuni*,*Yersinia* *enterocolitica*, *Salmonella* spp., *Shigella* spp., *E. coli* enterohemorrágica, Rotavirus *Entamoeba histolytica*, *Giardia lamblia*, Cestodos, Nemátodos.

El donador de heces deberá tener hemoglobina >12g/dl, enzimas AST y ALT no mayores a 2 veces el límite superior normal, tener ELISA vs Virus de la inmunodeficiencia humana (VIH) negativo, IgM de Virus de hepatitis A negativo, Antígeno s de Virus de hepatitis B negativo, VDRL negativo, IgM de Citomegalovirus negativo, anticuerpos vs virus de hepatitis C negativo. Criterios de exclusión: padre o madre con diabetes, embarazo, cirugías abdominales previas (excepto de pared abdominal, ejemplo: hernioplastías). Se eliminarán los sujetos que no deseen continuar en el estudio.

**2.4.2Preparación de muestras de microbiota fecal y almacenamiento**

Se obtendrán 150-200gr de heces del donador y se procesarán inmediatamente Todas las muestras serán etiquetadas para identificar apropiadamente al donador. Las heces se mezclarán con 500ml de solución salina al 0.85% en una mezcladora para obtener un fluido espeso. Se filtrará el fluido en una gasa estéril remover partículas biológicas mayores a 330 micras y se congelarán alícuotas de 250ml a -20°C hasta por 6 meses.

Cuando se requiera aplicar las muestras, se descongelarán 1 hora a baño maría (30°C). Se almacenarán alícuotas para fines de investigación en la misma línea de Infectología, no serán comercializadas, estudios moleculares y reevaluación en caso de eventos adversos.

**2.4.3Selección y seguimiento de pacientes**

Los pacientes se aleatorizarán por método de sobre cerrado 1:1 y se separarán en 2 grupos de tratamiento. El grupo 1 pacientes serán sometidos a trasplante de microbiota fecal por sonda nasogástrica o nasoyeyunal o en caso necesario por endoscopia y grupo 2 tratamiento con vancomicina 250mg vía oral o por sonda nasogástrica o nasoyeyunal cada 6 horas. Previo inicio de tratamiento se determinará el peso del sujeto y la glicemia preprandial, así como signos vitales, presencia de dolor abdominal, peristaltismo, número y características de evacuaciones de acuerdo a la escala de Bristol; posteriormente se evaluarán estos puntos cada 24 horas hasta egreso hospitalario, además a los 7, 14, 28 días 3 y 6 meses posteriores a tratamiento. Se determinarán signos vitales las primeras 4 horas posteriores al inicio de tratamiento, escala ATLAS, APACHE y SOFA basal, a las 24, 48 hrs y 5 días postratamiento.

Se les realizará cultivo para *C. difficile* al inicio, a los 14 días y 3 meses posteriores a tratamiento. Se determinarán la glicemia preprandial y el peso en los días 14, 28, 3 y 6 meses y se buscará intencionadamente la toma de medicamentos concomitantes antes del tratamiento y a los días 1, 7, 14, 28, 3 y 6 meses.

**2.4.4 Administración de trasplante de microbiota fecal**

Los pacientes que se incluyan en el grupo de trasplante de microbiota fecal serán sometidos a éste por una de las siguientes formas. Instilación directa de 250ml de las alícuotas preparadas tras su descongelación a través de sonda nasogátrica o nasoyeyunal o por vía endoscópica. Se instilarán la alícuotas en duodeno o yeyuno; se administrarán 20ml de alícuotas seguidos de 10ml de agua potable (total 30ml) cada 20 min hasta completar 100ml de alícuotas.

**2.5 Análisis estadístico**

Para el análisis de no inferioridad se incluirán 10 pacientes en cada grupo. Se utilizará el programa SPSS versión 15 y Excel. (Poder estadístico del 0.9356, error tipo α 5%, margen de no inferioridad o superioridad de -0.5, desviación estándar de 1).

Se contará con al menos 2 donadores sanos no relacionados al paciente para que en caso de no responder al primer trasplante de microbiota se realice un segundo trasplante con donador diferente.

**3. BIBLIOGRAFIA**

1. Mark A Miller, Thomas Louie, Kathleen, Karl Weiss, Arnold Lentnek, Yoav Golan, Yin Kean and Pam Sears; Derivation and validation of a Simple clinical bedside score (ATLAS) for Clostridium difficile infection which predicts response to therapy; BMC Infections Diseases 2013, 13:148.
2. Giovanni Cammarota, Gianluca Ianiro, Stefano Bibbó, Antonio Gasbarrini; Gut microbiota modulation: probiotics, antibiotics or fecal microbiota transplantation?; Inter Emerg Med, March 2014.
3. Johan S. Bakken et al. Treating *Clostridium difficile* Infection with fecal microbiota transplantation, Clinical Gastroenterology and hepatology 2011; 9:1044-1049
4. Derrick W. Crook et al., Fidaxomicin versus vancomycin for *Clostridium difficile* Infection: Meta-analysis of Pivotal randomized Controlled trials; Clinical infectious diseases 2012;55(S2):S93-103.
5. Edward C Oldfield IV et al; Clinical update for the diagnosis and treatment of *Clostridium difficile* infection; World J Gastrointest Pharmacol ther 2014 February 6;5 (1):1-26.
6. Sahil Khanna and Darrell S. Pardi; *Clostridium difficile* infection: management strategies for a difficult disease; Ther Adv Gastroenterol 2014 Vol 7 (2) 72-86.
7. Henrik Knecht et al Effects of β-lactam Antibiotics and Fluoroquinolones on Human Gut Microbiota in relation to *Clostridium difficile* associated Diarrhea; Plos One February 2014 Vol 9 Issues 2 e89417
8. Gauree G. Konijeti et al. Cost effectivness of Competing Strategies for management of Recurrent *Clostridium difficile* Infection: A decision Analysis; Clinical Infectious Diseases 2014;58 (11):1507-14
9. Daniel Merenstein, Najwa El-Nachef and Susan V. Lynch; Fecal Microbiota Theraphy- Promises and Pitfalls, Journal of Pediatric Gastroenterology and Nutrition Publish Ahead of Print
10. Stephanie M. Rabe, Treatment of recurrent *Clostridium difficile* infection With fecal Trasplantation, Society of Gastroenterology nurses and Associates Vol 37, Num 2, March/April 2014.
11. Giuseppe Russello Terapia delle infezioni da *Clostridium difficile* con trapianto di feci: controllli microbiologici sul donatore; La Infezioni in Medicina, n.1,5-10,2014.
