# Supplementary material for: Randomized clinical trial to evaluate the effect of fecal microbiota transplant for initial Clostridium difficile infection in intestinal microbiome
Source: PLoS One. 2017 Dec 20;12(12):e0189768. doi: 10.1371/journal.pone.0189768 (PMC5738078; doi:10.1371/journal.pone.0189768)
Supplement: S2 File — Translation of document protocol (English). (DOCX) [file pone.0189768.s002.docx]

**“INITIAL TREATMENT WITH FECAL MICROBIOTA TRANSPLANTATION IN PATIENTS WITH INFECTION BY *Clostridium difficile."***

1. **THEORETICAL FRAMEWORK**

*Clostridium difficile* infection (CDI) is a challenge in hospital-acquired infections, and has increased over the last decade and has even overcome methicillin-resistant *Staphylococcus aureus* (MRSA) infection as the leading cause of in-hospital infection. This infection is the most common cause of hospital diarrhea. Also, the emergence of a quinolone resistant strain of *C. difficile*, which produces 16 times more toxin A and 23 times more toxin B than other strains of *C. difficile*, has been reported.

The use of antibiotics increases the risk of CDI 8 to 10 times more in the following month and 3 times in the second month; there are multiple associations with the consumption of ampicillin, clindamycin and third generation cephalosporins, although the use of any antibiotic (including metronidazole) has been associated with the development of CDI. The use of multiple antibiotics during more than ten days has been related to increased risk to the development of CDI. However, exposure to antibiotics is not necessary for the acquisition of the disease, in these cases, the disease is associated with a close contact with patients with CDI. It has been reported that 25% of cases are in patient care homes.

Other factors associated with the development of CDI are the use of antineoplastic agents, which alter the fecal microbiota and are capable of inducing mitotic arrest in epithelial cells, causing necrosis and desquamation of the mucosa. Age is another risk factor associated with the development of CDI, with a 10-fold increased risk for patients 60-90 years old. It has been reported that 90% of deaths are in people over 65 years. Other factors associated with CDI include the use of enemas, gastrointestinal stimulants, and post pyloric enteral feeding, which increases the risk of developing CDI by 11-fold. Multiple studies have found a high risk in users of proton pump inhibitors, which increase the ability of *C. difficile* spores to convert to their vegetative form and to survive in the lumen of the gastrointestinal tract; even so, the multivariate analysis does not show significant differences versus the studies that showed direct association in the univariate analysis; so the prevention, detection, and prevention of CDI does not include use of proton pump inhibitors.

The clinical presentation of CDI varies from asymptomatic infection (with 20% of cases), colitis with or without pseudomembranes, to fulminant colitis and megacolon. The presence of dysenteric diarrhea is uncommon with only 5-10% of cases, fever in 30 to 50% of cases and only 26% have occult blood in feces. Leukocytosis is common in 50% to 60% of cases, and hypoalbuminemia is highly suggestive of CDI. Hypoalbuminemia is a result of a loss of protein by protein-losing enteropathy. Fecal leukocytes have been found in 28-40% of cases.

Endoscopic diagnosis is used when *C. difficile* A and B toxins cannot be determined or when they are negative with high clinical suspicion. Endoscopy usually reveals pseudomembranes, and in 22% of cases where they are observed macroscopically, they are not observed in histology. The neutralization and cytotoxic cell culture assay is partially accepted as the diagnostic gold standard, although it is expensive, not standardized and available only in research laboratories. The toxigenic culture is considered by some the method of choice. In culture, feces are cultured in a selective medium, subsequently, the organism is tested for the production of toxins. Enzyme-linked immunoassay determines the presence of toxins A and B, has a sensitivity of 38% and a positive predictive value of 50%. The lack of specificity of the A/B toxin by EIA has led to the search for more accurate methods for diagnosis. The detection of Glutamate dehydrogenase (GDH) is promising, it is an antigen commonly expressed in high levels by *C. difficile* strains, but only demonstrates the presence of *C. difficile*, not the presence of toxigenic strains (20% of the strains are nor toxigenic) nor the presence of toxins in faeces, so in case of positive GDH test would require a second examination and confirm the presence of toxins.

The use of polymerase chain reaction (PCR) to detect the toxin producing gene (*tcdB*), is rapid (approximately 2 hours) with a limit of detection of 105 copies/gr of feces, although the cost is 5 to 10 times higher than the determination of toxins and its sensitivity is 91%. However, with this test the toxin producing gene is detected, but not the toxin; so potentially asymptomatic carriers would be detected. Also, the test cannot be used for relapse cases since 56% of patients have this test positive within 1 to 4 weeks after finishing treatment. Therefore, it has a positive predictive value of 61%. Currently, the guidelines of the American College of Gastroenterology (ACG) recommend amplification of nucleic acids as PCR as the gold standard for diagnosis of CDI. They also suggest that GDH determination can be used as part of the diagnostic algorithm and confirmed with A/B toxin determination by EIA.

Treatment is controversial to date because it cannot establish the efficacy of antibiotic therapy, no single antibiotic is superior to another. The use of metronidazole is the antibiotic preferred by the development of *Enterococcus* resistant to vancomycin and the cost of oral vancomycin. Therefore, multiple centers have replaced vancomycin capsules with the use of the generic intravenous formulation of vancomycin reconstituted in water, used as a liquid enteral solution. In addition to the differences in cost and availability of metronidazole and vancomycin, no significant differences were found between the use of both antibiotics. Despite similar responses with the use of these drugs, there are pharmacological differences favoring vancomycin, since only 14% of metronidazole total is eliminated by feces, compared to vancomycin, since absorption is practically nil. Despite the low levels of metronidazole in vitro, it has been shown that fast bactericidal with only eight times the minimal inhibitory concentration (MIC). It has been shown that 41% of patients receiving antibiotic therapy fail to treatment within the first 14 days. Also, the role for rifampicin has been established as an adjuvant treatment with metronidazole.

The therapeutic decision is based on the severity of the clinical picture. According to the ACG's 2013 recommendations, they classify the severity as mild to moderate, severe and severe-complicated. The mild or moderate case is defined as diarrhea with some other symptomatology that does not meet criteria of severity, treatment with oral metronidazole (VO) is indicated, and in the case of lack of improvement in 5 to 7 days, a change to vancomycin is considered. In the case of a severe case that includes albumin <3 g/dl and leukocytosis, >15,000 cel/mm^3^ and abdominal pain the recommended treatment is vancomycin VO. Severe disease including admission to intensive care, hypotension with or without the use of vasopressors, fever >38.5°C, ileus or abdominal distension, altered mental status, leukocytosis >35,000 cells/mm^3^, or leukopenia <2000 cells/mm^3^, serum lactate >2.2 mmol/L or organic failure; the use of vancomycin VO, simultaneous to the rectal route and intravenous metronidazole is recommended.

The role of surgical treatment is reserved for cases that fail at the maximum medical management manifested as unresolved sepsis, cecal dilation greater than 10 cm (megacolon) or perforation of the intestine. With a reported incidence of 0.4-3.6% of the cases, of which the mortality varies from 30 to 80%. The optimal treatment at this point is total colectomy since, compared with hemicolectomy, mortality increases from 11% to 100%. One of the strongest predictors of mortality is the use of preoperative vasopressors, which increases 4-fold the risk of death.

Fecal microbiota transplantation has been used as a promising alternative to non-antibiotic therapy and has been used to date in patients who have experienced multiple relapses or recurrences. Usually, the fecal material to be transplanted is obtained from a related donor. It has been used in patients with CDI with a rapid response and without recurrence in 86%, with patients free of diarrhea in months or years. The results of this treatment vary in the different series with the type of antibiotics used before transplantation. This type of therapy has only been explored in refractory or recurrent cases. One of the major limitations is the alternative treatment as well as being aesthetically unpleasant. Despite this, in studies with patients treated with this method, 97% would receive treatment again, and 53% would have chosen it as the initial treatment.

An open, randomized, controlled study in Netherlands in 2013 (van Nood *et al*) used patients with relapse of ICD and compared the treatment by dividing patients into a 3 arm protocol comparing vancomycin plus microbiota transplantation in the first arm, in the second arm they used vancomycin and intestinal lavage and in the third arm conventional treatment with vancomycin was used. The findings were 94%, 23%, and 31% cure respectively. This demonstrates the therapeutic efficacy of fecal microbiota transplantation. One of the problems that could be faced when performing this type of transplant is the obtaining of stool samples for transplantation. The possibility of a sample bank for transplantation may be an accessible and fast strategy. This possibility was established when from 2004 to 2010 at the General Hospital of South Stockholm, stool transplants of samples obtained in 1994 from a single donor were performed.

**2. CLINICAL INVESTIGATION**

**2.1 Justification**

Mortality in patients with *Clostridium difficile* infection (CDI) is 20% and is higher in patients with severe infection. Fecal microbiota transplantation has a cure rate of >90% in patients with recurrent CDI. Fecal microbiota transplantation has not been studied as the first line of treatment in CDI**.**

**2.2 Hypothesis**

**2.2.1 True hypothesis**

Fecal microbiota transplantation is superior to antibiotic therapy as the first line of treatment in patients with *C. difficile* infection.

**2.2.2 Null hypothesis**

Fecal microbiota transplantation is not superior to antibiotic therapy as the first line of treatment in patients with *C. difficile* infection.

**2.3 Objectives**

**2.3.1 Primary objective**

To determine the therapeutic efficacy of fecal microbiota transplantation as the first line of treatment compared to the use of vancomycin enteric route in patients with *C. difficile* infection.

**2.4 Materials and methods**

**2.4.1 Criteria for selection of patients and donors**

Patients older than 18 years with a diagnosis of CDI will be included by any of the following methods: toxin detection by immunoassay, GeneXpert positive test, positive stool culture for *C. difficile*, a colonoscopic image suggestive of CDI. Patients who agree to participate in the study will be included by signed informed consent. Patients with toxic megacolon, suspected or documented intestinal perforation, pregnancy and the concomitant presence of colon neoplasms will be excluded. Patients who decide to leave the study will be eliminated.

Concerning the selection of donors, they will be inquired about the history of travel, sexual behavior, previous surgeries, transfusions and other risk factors. We will include healthy subjects, altruistic donors of blood, that allow access to confidential information obtained in Blood Bank of the University Hospital about their results of laboratory tests that will be requested from the Blood Bank and will be provided to the researcher's team, which will deal with the Confidential information. In addition, subjects should have a body mass index of 20 to 25 kg/m^2^, unrelated to patients, with no history of autoimmune diseases, absence of diabetes or metabolic syndrome, who have not received systemic antibiotics in the last 3 months, who have not received proton pump inhibitors in the 2 weeks prior to collection of the specimens, who have not received immunosuppressants in the past 12 months, who have no history of communicable infectious diseases, and who are free of communicable infectious diseases, have not had diarrhoea in the 3 months prior to the collection of the sample, who have not been hospitalized in the last 3 months and in the case of women, not pregnant donors. Stool specimens obtained from donors should be free of the following microorganisms: *C. difficile, Helicobacter pylori, Campylobacter jejuni, Yersinia enterocolitica, Salmonella* spp., *Shigella* spp., Enterohemorrhagic *E. coli*, Rotavirus, *Entamoeba histolytica*, *Giardia lamblia*, Cestodes, Nematodes. The donor should have hemoglobin >12g/dL, AST, and ALT enzymes not greater than two times the upper limit of normal, have ELISA vs. HIV negative, IgM from hepatitis A virus negative, the antigen from Hepatitis B virus negative, negative VDRL, cytomegalovirus-negative IgM, antibodies vs. hepatitis C virus negative. Exclusion criteria: father or mother with diabetes, pregnancy, previous abdominal surgeries (except abdominal wall, example: hernioplasty). Subjects that do not wish to continue in the study will be eliminated.

**2.4.2 Preparation of fecal microbiota samples and storage**

150-200 g of donor feces will be obtained and processed immediately. All samples will be labeled to identify the donor properly. The feces will be mixed with 500 ml of 0.85% saline solution in a mixer to obtain a thick fluid. The fluid will be filtered on a sterile gauze removing biological particles greater than 330 microns, and 250 ml aliquots will be frozen at -70°C for up to 6 months. When samples are required, they will be thawed for 1 hour in a water bath (30°C). Aliquots will be stored for research purposes in the same line of Infectology, molecular studies and reevaluation in the case of adverse events.

**2.4.3 Patient selection and follow-up**

Patients will be randomized by 1:1 closed envelope method and separated into two treatment groups. The group 1 patients will be submitted to fecal microbiota transplantation by nasogastric or nasojejunal tube or if necessary by endoscopy. Group 2 will be treated with vancomycin 250 mg orally or nasogastric or nasojejunal tube every 6 hours. Prior to initiation of treatment, the subject's weight and preprandial blood glucose, as well as vital signs, presence of abdominal pain, peristalsis, number and characteristics of bowel movements according to the Bristol scale will be determined, then these points will be evaluated every 24 hours until hospital discharge, in addition to 7, 14, 28 days 3 and 6 months after treatment. Vital signs will be determined the first 4 hours after the start of treatment, ATLAS, APACHE and SOFA at baseline, at 24, 48 hrs and five days posttreatment.

Cultivation will be performed for *C. difficile* at the beginning, at 14 days and three months after treatment. Preprandial glycemia and weight will be determined on days 14, 28, 3 and six months, and the intention to take pre-treatment medication on days 1, 7, 14, 28, 3 and six months will be determined.

**2.4.4 Administration of fecal microbiota transplantation**

Patients who are included in the fecal microbiota transplantation group will be submitted to one of the following forms: A) 250 ml of direct instillation of aliquots prepared after thawing through nasogastric or nasojejunal tube or endoscopically, B) the aliquots will be instilled in the duodenum or jejunum; 20 ml of aliquots followed by 10 ml of drinking water (total 30 ml) every 20 minutes until 100 ml of aliquots is completed.

**2.5 Statistic analysis**

For the non-inferiority analysis, ten patients will be included in each group. The program SPSS version 15 and Excel will be used (Statistical Power of 0.9356, error type 5%, the margin of non-inferiority or superiority of -0.5, a standard deviation of 1). There will be at least two healthy donors not related to the patient so that in the case of not responding to the first transplant of microbiota a second transplant with a different donor will be performed.

**3. BIBLIOGRAPHY**

1. Mark A Miller, Thomas Louie, Kathleen, Karl Weiss, Arnold Lentnek, Yoav Golan, Yin Kean and Pam Sears; Derivation and Validation of a Simple clinical bedside score (ATLAS) for Clostridium difficile infection which predicts response to therapy; BMC Infections Diseases 2013, 13:148.
2. Giovanni Cammarota, Gianluca Ianiro, Stefano Bibbó, Antonio Gasbarrini; Gut microbiota modulation: probiotics, antibiotics or fecal microbiota transplantation?; Inter Emerg Med, March 2014.
3. Johan S. Bakken et al. Treating *Clostridium difficile* Infection with fecal microbiota transplantation, Clinical Gastroenterology and hepatology 2011; 9:1044-1049
4. Derrick W. Crook et al., Fidaxomicin versus vancomycin for *Clostridium difficile* Infection: Meta-analysis of Pivotal randomized Controlled trials; Clinical infectious diseases 2012;55(S2):S93-103.
5. Edward C Oldfield IV et al.; Clinical update for the diagnosis and treatment of *Clostridium difficile* infection; World J Gastrointest Pharmacol Ther 2014 February 6;5 (1):1-26.
6. Sahil Khanna and Darrell S. Pardi; *Clostridium difficile* infection: management strategies for a difficult disease; Ther Adv Gastroenterol 2014 Vol 7 (2) 72-86.
7. Henrik Knecht et al. Effects of β-lactam Antibiotics and Fluoroquinolones on Human Gut Microbiota in relation to *Clostridium difficile* associated Diarrhea; Plos One February 2014 Vol 9 Issues 2 e89417
8. Gauree G. Konijeti et al. Cost effectiveness of Competing Strategies for management of Recurrent *Clostridium difficile* Infection: A decision Analysis; Clinical Infectious Diseases 2014;58 (11):1507-14
9. Daniel Merenstein, Najwa El-Nachef and Susan V. Lynch; Fecal Microbiota Theraphy- Promises and Pitfalls, Journal of Pediatric Gastroenterology and Nutrition Publish Ahead of Print
10. Stephanie M. Rabe, Treatment of recurrent *Clostridium difficile* infection With fecal Trasplantation, Society of Gastroenterology nurses and Associates Vol 37, Num 2, March/April 2014.
11. Giuseppe Russello Terapia delle infezioni da *Clostridium difficile* con trapianto di feci: controllli microbiologici sul donatore; La Infezioni in Medicina, n.1,5-10,2014.
